# Effect of olive by-products feed supplementation on physicochemical and microbiological profile of Provola cheese

**DOI:** 10.3389/fmicb.2023.1112328

**Published:** 2023-01-16

**Authors:** Francesco Maria Calabrese, Nunziatina Russo, Giuseppe Celano, Alessandra Pino, Vincenzo Lopreiato, Federica Litrenta, Giuseppa Di Bella, Luigi Liotta, Maria De Angelis, Cinzia Caggia, Cinzia L. Randazzo

**Affiliations:** ^1^Department of Soil, Plant and Food Science, University of Bari Aldo Moro, Bari, Italy; ^2^Department of Agricultural, Food and Environment, University of Catania, Catania, Italy; ^3^ProBioEtna SRL, Spin-off of University of Catania, Catania, Italy; ^4^Department of Veterinary Sciences, University of Messina, Messina, Italy; ^5^Department of Biomedical, Dental and Morphological and Functional Imaging Sciences, University of Messina, Messina, Italy

**Keywords:** microbiota, PUFA, volatilome, dairy products, olive cake

## Abstract

**Introduction:**

With the purpose to evaluate the effects of dietary olive cake, a source of bioactive phenolic compounds, as feed supplementation of lactating dairy cows on fatty acid composition, volatile organic compounds, and microbiological profiles of Provola cheese, we performed a two-arm study where control and experimental administered cows derived dairy have been compared.

**Methods:**

Our panel of analyses include metabolomics, physicochemical detected variables, culture dependent and independent analyses, and a stringent statistical approach aimful at disclosing only statistically significant results.

**Results and discussion:**

Looking at the physicochemical variable’s profiles, a higher content of unsaturated fatty acids, polyunsaturated fatty acid, and conjugated linoleic acids as well of proteins were observed in experimental cheese samples, indicating the beneficial effect of dietary supplementation. Furthermore, based on volatilome composition, a clear cluster separation between control and experimental cheeses was obtained, mainly related to terpenes degradation, able of influencing their aroma and taste. Microbiological results showed a decrease of some spoilage related microbial groups in experimental cheeses, probably due to the inhibitory effect exerted by polyphenols compounds, that contrarily did not affect the core taxa of all cheese samples. This paper confirmed the promising utilization of olive by-product in farming practices to obtain more sustainable and safe dairy food products with lower environmental impact, mainly in Sicily and Mediterranean area, where waste disposal poses serious environmental and economic problems.

## 1. Introduction

To date, several studies, performed in the sector of ruminant husbandry, showed the crucial role of feeding strategies to influence the chemical and nutritional profiles of animal-based foods (Bennato et al., 2019). In particular, the use of agro-industrial by-products, as feed supplements, along preserving animal welfare (Iannaccone et al., 2019), can improve the nutritional quality of animal products (Chedea et al., 2016; Ianni and Martino, 2020), inducing, especially in dairy foods, the development of volatile organic compounds which influence aroma and taste (Bennato et al., 2020; Ianni and Martino, 2020). Furthermore, the need to both solve the problem of the disposal of waste material and reduce production costs for livestock feeding made the use of agro-industrial by-products the best strategy to undertake (Caputo et al., 2015; Milani et al., 2020). In Sicily and the Mediterranean area, an issue of great importance is related to the reuse of waste biomass derived from the production of olive oil, as a suitable strategy to overcome both environmental and economic problems (Awawdeh and Obeidat, 2011; Cibik and Keles, 2016; Chiofalo et al., 2020; Liotta et al., 2020; Foti et al., 2021, 2022). Several studies focused on the possible use of these by-products as feeding supplements for farm animals, both ruminant and monogastric (Ishfaq et al., 2015; Kotsampasi et al., 2017; Mannelli et al., 2018; Biondi et al., 2019; Dorbane et al., 2019; Liotta et al., 2019).

Olive cake, the main by-product of the olives industry, accounting for about 50% of the conversion process (Caputo et al., 2015), represents an economic raw material that can be used as a source of bioactive phenolic compounds, such as oleuropein, hydroxytyrosol, verbascoside, apigenin-7-glucoside, and luteolin-7-glucoside, with antioxidant, antihypertensive, and anti-inflammatory functions (Özcan and Matthäus, 2016; O’Callaghan et al., 2017; Bennato et al., 2020). However, in the past, the inclusion of olive cake in ruminant nutrition was negatively considered as low-quality feedstuff for the digestibility of the organic matter, related to the high ligno-cellulotic content (Molina-Alcaide and Yáñez Ruiz, 2008) and low protein and energy (Awawdeh and Obeidat, 2013). Currently, modern extraction technologies allow to reduce, totally or partially, the presence of the seeds (stoning) and therefore the lignin content, increasing feed palatability (Caputo et al., 2015). The major aim of polyphenols inclusion in ruminant nutrition is the reduction of biohydrogenation of polyunsaturated fatty acids (PUFA), the increase of the accumulation of vaccenic acid (VA) in the rumen and of fatty acids in the mammary gland, and the consequent increase of PUFA, VA, and conjugated linoleic acid (CLA; derived from the enzymatic desaturation of VA) amount in the final product, with an improvement of food quality (Serra et al., 2021). Although it is well known that diet, through the rumen metabolisms action, provides the valorization of the chemical characteristic of this bio-waste, improving the fatty quality of dairy products (Mannelli et al., 2018; Chiofalo et al., 2020), studies related to the alteration of ruminal biohydrogenation process and modifications milk fatty acids profile provide not univocal results. Bennato et al. (2020) widely demonstrated, in lactating ruminants, the influence of diet on volatile profile of dairy products, both fresh and ripened. Other studies showed a significant modification in the fatty acid composition and sensory profile of the milk, while several trials reported no modification in the milk yield and in the animal performance (Terramoccia et al., 2013; Mele et al., 2014; Cibik and Keles, 2016; Chaves et al., 2021) and the aromatic profile animal-derived food (Caputo et al., 2015). Therefore, it is of paramount importance to in depth investigate the relationship between feeding management strategies and nutritional and nutraceutical properties of dairy products, in order to meet the current trend of consumer choices toward high-quality food (Bonanno et al., 2019).

Considering the biological relevance of olive by-product in animal feeding, as an interesting strategy to both minimize their eco-footprint and improve the quality of animal-derived products, the purpose of this work was to evaluate the effects of olive cake feed supplementation on fatty acid composition, volatile organic compounds, and microbiological profiles of Provola cheese.

## 2. Materials and methods

### 2.1. Ethical statement

All procedures were conducted according to the European guidelines for the care and use of animals in research (Directive 2010/63/EU). The experimental protocol was approved by the Ethical Committee of the Department of Veterinary Science of the University of Messina (code 041/2020).

### 2.2. Animals and diets

The experimental period lasted from February to June 2021 and was performed on a commercial dairy farm located in the Sicilian region (Ragusa, Italy) at an altitude of 520 m above sea level. One hundred and twenty healthy multiparous dairy Friesian cows were divided into two groups (60 animals each), called Control (Ctr) and Experimental (Exp), homogenous for the Body Condition Score (3 ± 0.5), distance from calving (90–120 days), and milk production (25 ± 3 kg/day), fed a diet (20 kg of Dray Matter (DM)/head per day as Total Mixed Ration) with concentrate (Crude Protein: 170 g/kg of DM; Crude Cellulose 69 g/kg; Crude oils and fats: 4.1 g/kg of DM; Ashes 76 g/kg of DM) and meadow hay (Crude Protein: 110.9 g/kg of DM; Ether Extract: 25.0 g/kg of DM; Neutral Detergent Fiber: 521.9 g/kg of DM). The Control group (Ctr) received a concentrate without any olive cake supplements, whereas the Experimental group (Exp) received a concentrate supplemented with the olive cake as 8% on a DM as reported in Table 1. A 3-week period of adaptation to diets was assured before collecting samples. The by-product used was represented by the residue of the mechanical pressing of olives with the two-stage process, to produce extra virgin olive oil. In addition, the by-product contained about 5% of leaf residue with which the olives normally arrive at the mill. This olive cake was subsequently pitted by centrifugation and dried in the open air. Its moisture content went from 60% to a value of 10%. Its nutritional characteristics are reported in Table 2.

**Table 1:** Diet ingredient composition (g kg^−1^ as fed).

Ingredient	Ctr	Exp
Cornmeal	39.00	38.00
Soybean meal (0.48 CP^*^)	19.00	18.00
Wheat middling	15.00	14.00
Barley meal	12.00	10.00
Olive cake	–	8.00
Sunflower meal	6.00	5.00
Carob pulp	3.00	2.00
Beet pulp	3.00	2.00
Vitamin premix^**^	3.00	3.00

*CP, Crude Protein.

** Providing per kg of diet: 32,000 U vitamin A, 3200 U vitamin D3, 120 mg vitamin E, 8 mg vitamin B1, 1.6 mg vitamin B2, 0.016 mg vitamin B12, 400 mg niacin, 4 mg pantothenic acid, 400 mg choline chloride.

**Table 2:** Chemical, fatty acids composition and polyphenols content of the dried and pitted olive cake.

	Dried, pitted olive cake
g/kg dry matter	
Moisture	36.47 ± 0.57
Ashes	34.13 ± 0.81
Crude fat	180.80 ± 0.26
Energy (ME) (Kcal/Kg)	3535.1
Crude protein (total N × 6.25)	61.00 ± 1.73
Neutral detergent fiber	410.33 ± 0.20
Acid detergent fiber	320.53 ± 0.34
Total polyphenols	10.18 ± 1.59
Fatty acid (g/100 g fat)	
C14:0	0.02 ± 0.01
C16:0	16.14 ± 0.17
C16:1	0.64 ± 0.07
C17:0	0.32 ± 0.07
C17:1	0.32 ± 0.02
C18:0	3.80 ± 0.16
C18:1n-9	66.63 ± 0.19
C18:2	10.66 ± 0.16
C18:3	0.57 ± 0.05
C20:0	0.53 ± 0.05
C20:1	0.33 ± 0.03
C22:0	0.03 ± 0.01
C24:0	0.01 ± 0.01

### 2.3. Milk samples and cheese making

Milk samples, obtained from control and experimental groups, were monthly collected in distinct tanks after mechanical milking performed in the evening (3 p.m.) and in the morning (7 a.m.). Samples were transferred, under refrigerated conditions (4 ± 2°C), to the Natura & Qualità (Soc. Agr. ARL) dairy company (Ragusa, Italy) and subjected to both physicochemical and microbiological analyses before cheese-making. Milk samples were analyzed for the fat, protein, casein, and lactose content by using a Fourier transform infrared (Milkoscan FT2, FOSS, Hillerød, Denmark) and the somatic cell count was determined using a FossomaticTM FC (FOSS, Hillerød, Denmark). Microbiological count was performed to assess the presence of *Enterobacteriaceae*, *Escherichia coli* and coliforms, total mesophilic bacteria, and *Listeria monocytogenes*, using the following agar media and growth conditions: Violet Red Bile Agar (VRBA) anaerobically incubated at 37°C and 45°C for 24–48 h, for the count of Enterobacteria and fecal coliforms, respectively; Plate Count Agar (PCA), aerobically incubated at 30°C for 48–72 h, for total mesophilic aerobic bacteria count; and Chromatic ECX gluc agar aerobically incubated at 37°C for 18–24 h for *E. coli.* In addition, according to the International Organization for Standardization (ISO), a two-stage enrichment method for *L. monocytogenes* (ISO (International Organization for Standardization) (ISO 11290-1:2017), 2017a) and *Salmonella* spp. detection (ISO (International Organization for Standardization) (ISO 6579-1:2017), 2017b) was used. All media were purchased from Liofilchem (Roseto degli Abruzzi, Italy).

**Figure 1:**
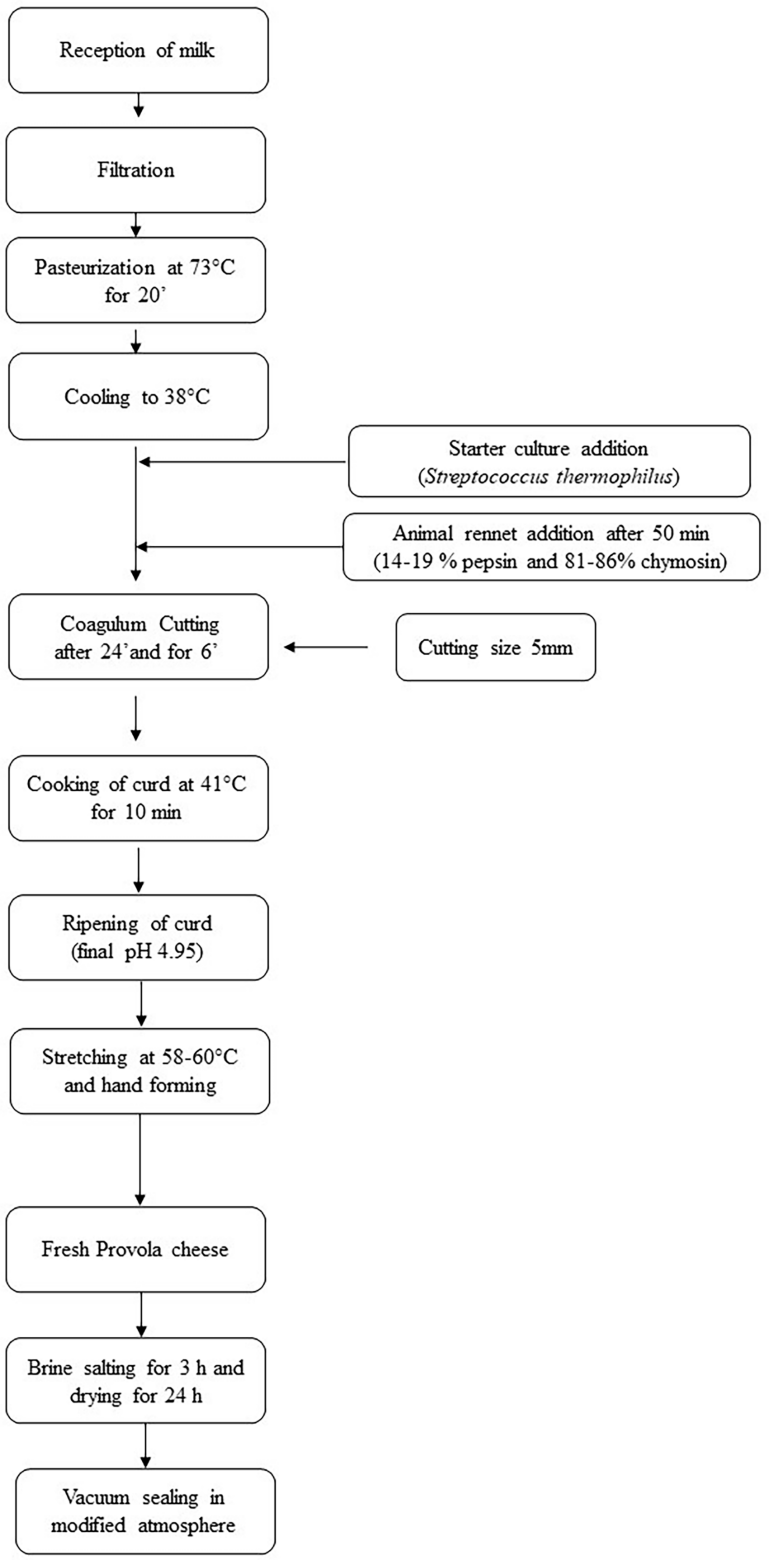
Provola cheese flowchart. Step by step followed flowchart useful for Provola cheese production.

Cheese samples (weight 500 g) were obtained following the flowchart as reported in Figure 1. Provola cheese samples, from each group, were monthly collected, from March to July, vacuum-packed, and transported under refrigerated conditions (4 ± 2°C) to the Department of Veterinary Sciences of the University of Messina for nutritional analysis, and to the Department of Agriculture, Food and Environment, University of Catania for microbiological and 16S rRNA gene meta-taxonomic analysis.

### 2.4. Cheese physicochemical analysis

One-day-old Provola cheese samples (100 g) were analyzed for chemical composition by FoodScan™ Dairy Analyser (FOSS, Italy). The fatty acid methyl esters (FAME) of cheese were analyzed by a GC–FID. For the chromatographic analyses, on 15 mg of lipids of cheese, the fatty acids methyl esters were prepared by using a solution of sulfuric acid/methanol (1:9, v/v) and submitted to a HRGC analysis. A gas chromatograph with a FID detector (Agilent Technologies 6890N, Palo Alto, CA, United States) equipped with a SP-2560 fused silica capillary column (100 m × 0.25 mm i.d. × 0.2 μm film thickness, Supelco, Inc., Bellefonte, PA, United States) was used. Helium was the carrier gas, 1 mL/min. The volume of the injection was 1 μL and the split ratio 1:50. The column temperature was programmed: an initial isotherm of 70°C (2 min), an increment of 15°C/min to 155°C (held for 25 min), then an increment of 3°C/min and a final isotherm of 215°C (8 min), following the procedure described by Tudisco et al. (2015). The fatty acids were identified by comparing the relative retention times of FAME peaks from samples containing standards from Supelco (mix 37 FAMEs, Supelco, Bellefonte, PA, United States). Chromatogram peak areas were acquired and calculated using a ChemStation software (Agilent, Santa Clara, CA, United States). The concentration of each fatty acid was expressed as g/100 g, considering 100 g as the summation of the areas of all the FAME identified. For each sample, the chromatographic analysis was repeated three times. The total polyphenol content was determined spectrophotometrically using the Folin–Ciocalteu’s reagent according to the method of Shetty et al. (1995). Approximately 50 mg of homogenized Provola was weighed, and 2.5 mL of 95% ethanol was added and kept at 0°C for 48 h. Around 1 mL of the supernatant was transferred into a tube and mixed with ethanol (95%) and ultrapure water. Folin–Ciocalteu’s reagent (50%) and Na_2_CO_3_ (5%) were added. Absorbances were read at 760 nm with 95% ethanol as analytical blank, using a UV–visible spectrophotometer (UV-2401PC Shimadzu). A calibration curve was constructed using appropriate dilutions of a gallic acid standard solution (95% in ethanol). Determinations were performed in triplicate together with blank solutions.

### 2.5. Microbiological analysis and DNA isolation

Microbiological analysis of 1-day old control and experimental cheese samples, monthly collected from March to July, was performed according to Commission Regulation (EC) No 2073/2005 of 15 November 2005 On microbiological criteria for foodstuffs (n.d.) by sampling plans with five random collected samples. In detail, from each cheese sample, both core and surface sections (25 g) were sampled and treated as previously described (Pino et al., 2018). Microbiological count was performed according to Pino et al. (2021) and Randazzo et al. (2021) using the following agar media and conditions: Kanamycin Aesculin Azide (KAA) Agar, aerobically incubated at 37°C for 24–48 h, for enterococci count; Mannitol Salt Agar (MSA), incubated at 32°C for 48 h, for staphylococci count; M17, supplemented with 5 g/L of lactose, incubated at 30°C for 24–48 h, for lactococci; de Man Rogosa and Sharpe agar (MRS), adjusted to pH 5.4, incubated at 32°C for 72 h under microaerophilic condition, for lactobacilli; and Sabouraud Dextrose Agar (SDA), supplemented with chloramphenicol (0.05 g/L) incubated at 25°C for 3–5 days, for yeasts and molds count. VRBA and PCA were used as previously described for Enterobacteria, fecal coliforms, and for total mesophilic aerobic bacteria count. *Listeria monocytogenes* (ISO (International Organization for Standardization) (ISO 11290-1:2017), 2017a) was enumerated as previously reported. All media were purchased from Oxoid (Basingstoke, United Kingdom). Moreover, according to the International Organization for Standardization (ISO), a two-stage enrichment method for *Salmonella* spp. detection (ISO (International Organization for Standardization) (ISO 6579-1:2017), 2017b) was used.

Control and experimental cheeses were subjected to total bacterial DNA isolation using the DNeasy^®^ mericon^®^ Food kit (Qiagen, Germany) following the manufacturer’s instructions. DNA concentration was determined using the fluorimeter Qubit 4.0 (Invitrogen, Carlsbad, CA, United States) before storing at −20°C until use.

Results of microbiological analysis were calculated as mean values of three determinations and standard deviations and were expressed as log cfu/g.

### 2.6. 16S rRNA gene meta-taxonomic analysis

The DNA isolated from cheese samples was subjected to 16S rRNA gene meta-taxonomic analysis. In detail, the V3 region of the 16S rRNA gene sequence was amplified using the Probio_Uni e/Probio_Rev primer pair (Milani et al., 2013) and subjected to MiSeq (Illumina) by GenProbio srl (Parma, Italy). Bioinformatics analysis was performed as reported by Vaccalluzzo et al. (2022). 16S rRNA raw data from the study were deposited at NCBI Sequence Read Archive (SRA^1^) under accession code PRJNA 903126.

### 2.7. Analysis of cheeses volatile organic compounds

Four grams of grated cheese were added with 10 μL of internal standard solution (2-octanol, at 10 ppm), placed into 20 mL glass vials, and sealed with polytetrafluoroethylene-coated silicone rubber septa (20 mm diameter; Supelco, Bellefonte, PA, United States). To obtain the best extraction efficiency, the micro-extraction procedure was performed as described by Salum et al. (2017), with slight modifications. After sample equilibration (10 min at 54.75°C), a conditioned 50/30 μm DVB/CAR/PDMS fiber (Supelco, Bellefonte, PA, United States) was exposed for 60 min. The temperature was kept constant during analysis, and the vials were maintained on a heater plate (CTC Analytics, Zwingen, Switzerland) of a CombiPAL system injector Autosampler (CTC Analytics). The extracted VOC were desorbed in splitless mode (3 min at 220°C) and analyzed through a Clarus 680 (Perkin Elmer) gas-chromatography (GC) system equipped with a capillary Rtx-WAX column (30 m × 0.25 mm i.d., 0.25 μm film thickness; Restek, Bellefonte, PA, United States). The column temperature was set initially at 35°C for 8 min, then increased to 60°C at 4°C min^−1^, to 160°C at 6°C min^−1^, and finally to 200°C at 20°C min^−1^ and held for 15 min. Helium was used as the carrier gas at flow rate of 1 mL min^−1^. A single quadrupole mass spectrometer (MS) Clarus SQ 8C (Perkin Elmer) was coupled to the GC system. The source and transfer line temperatures were kept at 250 and 230°C, respectively. Electron ionization masses were recorded at 70 eV in the mass-to-charge ratio (m/z) interval 34–350 (Randazzo et al., 2021). Each chromatogram was analyzed for peak identification using the NIST (National Institute of Standard and Technology) 2008 library. A peak area threshold of 1,000,000 and at least 85% probability of match were used for dentification, followed by visual inspection of the fragment patterns when required. The concentrations of VOC (calculated on internal standard base) were expressed as mg kg^−1^.

### 2.8. Statistical analysis

With the aim of searching for statistically significant changes in alpha and beta diversity estimates and starting from Bray-Curtis, Jaccard, Weighted UniFrac, Unweighted UniFrac distance matrices, QIIME II nested plugins were used. The PERMANOVA test was computed in same software environment.

Taxa abundance in the two compared groups was inspected by mean of a compositionality-aware test, i.e., the Analysis of Composition of Microbiomes test (ANCOM), but also with the White’s non-parametric test corrected for multiple tests. In the case of White’s test, results have been shown in terms of boxplot.

For VOC statistical analyses, we stratified samples based on fed group and sampled month by make use of Partial least square discriminant analysis (PLSDA) and Orthogonal Projections to Latent Structures Discriminant Analysis (OPLS-DA).

## 3. Results

### 3.1. Milk and cheese analysis

Physicochemical composition and microbiological parameters of milk samples are reported in Supplementary Tables 1, 2, respectively. Cheese composition in terms of physicochemical parameters in both control and experimental samples, collected from March to July, is reported in Supplementary Table 3. The experimental cheese showed a higher content of polyphenols, proteins, n-3, n-6, PUFA, C18:0, C18:1n-9, C18:2n-6 cis, and C18:2n-6 trans, than control ones, whereas the concentrations of C14:0, C16:0, and C20:3n-3 were higher in the control cheeses. Physicochemical cheese detected variables were inspected for their differential abundances in the two compared groups in a Wilcoxon rank-sum test. Table 3 reports only parameters that were significantly different (FDR < 0.05).

**Table 3:** Cheese physicochemical parameter statistics.

FoodScan feature	Exp: mean rel. Freq. (%)	Exp: std. dev. (%)	Ctr: mean rel. Freq. (%)	Ctr: std. dev. (%)	Values of *p*	Values of *p* (corrected)	Difference between means	95.0% lower CI	95.0% upper CI
Atherogenic_index	0.5733	0.0283	0.6464	0.0303	0.0000	0.0000	−0.0731	−0.0924	−0.0539
C10:0	0.6047	0.0457	0.6725	0.0445	0.0000	0.0003	−0.0678	−0.0974	−0.0382
C12:0	0.6705	0.0608	0.7461	0.0681	0.0009	0.0034	−0.0756	−0.1180	−0.0332
C14:0	2.1492	0.1241	2.3633	0.1253	0.0000	0.0001	−0.2141	−0.2960	−0.1322
C14:1	0.1884	0.0090	0.2169	0.0194	0.0000	0.0001	−0.0285	−0.0386	−0.0184
C15:0	0.2285	0.0188	0.2550	0.0183	0.0001	0.0005	−0.0264	−0.0386	−0.0142
C16:0	5.9090	0.2621	6.7027	0.2556	0.0000	0.0000	−0.7937	−0.9637	−0.6237
C16:1	0.3075	0.0315	0.3424	0.0230	0.0004	0.0018	−0.0348	−0.0530	−0.0166
C18:0	2.4674	0.4441	2.0899	0.2168	0.0025	0.0083	0.3775	0.1451	0.6099
C18:3_n-3	0.0960	0.0213	0.0820	0.0089	0.0142	0.0339	0.0140	0.0030	0.0249
C20:1_n-9	0.0113	0.0022	0.0097	0.0019	0.0205	0.0446	0.0016	0.0003	0.0030
C20:3_n-3	0.0332	0.0120	0.0416	0.0044	0.0082	0.0215	−0.0085	−0.0145	−0.0024
C22:1_n-9	0.0032	0.0010	0.0022	0.0006	0.0012	0.0043	0.0009	0.0004	0.0015
C23:0	0.0062	0.0013	0.0074	0.0015	0.0124	0.0311	−0.0012	−0.0021	−0.0003
C24:0	0.0090	0.0011	0.0105	0.0017	0.0036	0.0107	−0.0015	−0.0024	−0.0005
C8:0	0.2494	0.0274	0.2918	0.0249	0.0000	0.0001	−0.0425	−0.0597	−0.0253
Humidity %	8.0827	0.7481	8.6942	0.2703	0.0027	0.0083	−0.6115	−0.9882	−0.2348
Polyphenols (ppm)	25.0441	3.6842	20.3088	2.5808	0.0001	0.0004	4.7353	2.6381	6.8324
∑_MUFA	4.3506	0.2993	4.6472	0.3098	0.0047	0.0132	−0.2966	−0.4967	−0.0965
∑_SFA	13.0742	0.6874	13.9585	0.4523	0.0000	0.0003	−0.8843	−1.2684	−0.5002
∑_tot	18.2166	0.9602	19.3793	0.6933	0.0001	0.0007	−1.1627	−1.7146	−0.6109
Total_fat	3.9284	0.4260	4.2976	0.5077	0.0202	0.0458	−0.3692	−0.6773	−0.0611
Thrombogenic_index	0.6896	0.0553	0.7468	0.0270	0.0004	0.0017	−0.0572	−0.0862	−0.0283

Cheese physicochemical parameter distribution was evaluated in Ctr and Exp samples. The two groups were compared by mean of a Wilcoxon rank-sum test and only False discovery rate (FDR) corrected variables were reported.

### 3.2. Cultivable microbiota

Results of microbiological analysis carried out on both control and experimental cheese samples are shown in Table 4. Overall, *Salmonella* spp., *L. monocytogenes*, *E. coli*, fecal coliforms, and molds were never detected in the analyzed samples, except for the control cheese collected in July, where coliforms reached the value of 2.00 log cfu/g (data not shown). Lactic acid bacteria (LAB) achieved an average value of about 6.84 log cfu/g in control samples and of 7.19 log cfu/g in experimental cheeses. In detail, control samples reached the highest values (7.99 log cfu/g) in April and the lowest (6.04 log cfu/g) in June, while the experimental ones achieved the highest (7.96 log cfu/g) in April and the lowest (6.55 log cfu/g) in July (Table 4). The lactococci group showed the same average value (about 8.00 log cfu/g) in both samples, while marked fluctuations were revealed during sampling period in the counts of enterococci, Enterobacteriaceae and coagulase-positive staphylococci groups (Table 4), with the lowest average value always found in the experimental samples. Significant differences (*p* ≤ 0.05) among control and experimental samples were revealed for total mesophilic bacteria in April and May, while the yeast count showed similar values in both samples, reaching average value of 5.17 log cfu/g (Table 4).

**Table 4:** Microbial counts of cheese samples.

Microbial groups	March		April		May		June		July		Mean	
	Ctr	Exp	Ctr	Exp	Ctr	Exp	Ctr	Exp	Ctr	Exp	Ctr	Exp
*Enterococcus* spp.	<1	<1	3.00 ± 0.06^a^	2.43 ± 0.18^b^	<1	<1	2.96 ± 0.17^a^	<1^b^	4.69 ± 0.25^a^	2.93 ± 0.04^b^	2.13 ± 2.05^a^	1.07 ± 1.48^b^
*Enterobacteriaceae*	2.61 ± 0.48^b^	3.35 ± 0.49^a^	5.08 ± 0.13^a^	3.66 ± 0.12^b^	2.04 ± 0.06	2.13 ± 0.18	<1^b^	2.66 ± 0.08^a^	2.70 ± 0.13^a^	<1^b^	2.49 ± 1.81	2.36 ± 1.45
Coagulase-positive staphylococci	<1^b^	2.39 ± 0.75^a^	2.31 ± 0.01^a^	<1^b^	<1	<1	2.84 ± 0.01^b^	4.08 ± 0.13^a^	3.16 ± 0.97^a^	<1^b^	1.66 ± 1.55	1.29 ± 1.87
Total mesophilic bacteria	2.81 ± 0.10	3.33 ± 0.47	5.93 ± 0.03^a^	5.06 ± 0.41^b^	6.62 ± 0.16^a^	5.74 ± 0.12^b^	4.84 ± 0.04^b^	8.03 ± 0.08^a^	6.59 ± 1.57	7.20 ± 0.01	5.35 ± 1.61	5.87 ± 1.84
Yeasts	4.43 ± 0.06	4.39 ± 0.56	6.67 ± 0.21	6.24 ± 0.09	3.51 ± 0.05	3.20 ± 0.28	4.80 ± 0.03	5.07 ± 0.15	6.61 ± 0.38	6.78 ± 0.02	5.20 ± 1.41	5.14 ± 1.43
*Lactococcus* spp.	6.92 ± 0.18	7.33 ± 0.02	8.77 ± 0.10	8.86 ± 0.12	6.96 ± 0.05^b^	7.70 ± 0.24^a^	8.79 ± 0.01^b^	8.68 ± 0.16^a^	8.73 ± 0.25^a^	7.47 ± 0.06^b^	8.03 ± 0.79	8.01 ± 0.63
Lactic Acid Bacteria	6.95 ± 0.07	7.16 ± 0.37	7.99 ± 0.02	7.96 ± 0.02	6.76 ± 0.07^b^	7.47 ± 0.09^a^	6.04 ± 0.06^b^	6.80 ± 0.07^a^	6.44 ± 0.01	6.55 ± 0.16	6.84 ± 0.65^b^	7.19 ± 0.50^a^

Data are presented as mean log cfu/g ± standard deviation, based on 3 replicates. Different superscript letters within the same line indicate significant differences at *p* < 0.05 between control and experimental samples for each sampling month.

### 3.3. Read statistics and taxonomic annotation

High-throughput sequencing output determined the relative abundance of bacterial species in Provola cheese samples esteemed based on denoised and taxonomically assigned reads.

A total of 496,430 reads from 10 cheese samples were retained after denoising steps. Sample metadata, denoising statistics, the taxonomy classification, and the relative abundances for each taxonomic level are reported in Supplementary File 1.

The mostly abundant genus was *Streptococcus,* which accounts for the great majority of relative abundance (99%) and the contribution of other detected taxa was very low. Noteworthy, among those genera that contributed with abundance percentages lower than 1%, *Lactobacillus, Lactococcus*, and *Acinetobacter* were found in all samples and can thus be considered as “core” taxa, whereas unclassified genera belonging to the family of Moraxellaceae and Chromohalobacter were not evenly distributed. Specifically, the *Chromohalobacter* genus was only detected in the experimental sample gathered in June. The great majority of taxa resulted not statistically significant in the performed Welch’s statistical test. The only one statistically significant difference found at the phylum level was between the control and treated groups sampled during the summer seasons. Specifically, although without a statistical significance after multiple test correction, Proteobacteria abundance decreased in experimental samples with respect to controls (Supplementary Figure S1). No other significant taxa were found when other taxonomic levels were inspected.

### 3.4. Alpha and beta diversity measures

No statistically significant differences were observed in alpha diversity index when control and experimental groups were compared, i.e., the two groups have comparable distributions both in Shannon and Faith’s PD metrics. Beta diversity distances including Bray-Curtis, Jaccard, and Weighted and Unweighted UniFrac distances did not showed divergent groups. The PCoA plot based on the unweighted UniFrac computed distances reveal no clustering. The pairwise PERMANOVA statistic based on Bray-Curtis distance values returned absence of statistical significance (Supplementary Figure S2).

### 3.5. Statistically significant volatile organic compounds and FoodScan detected physicochemical variables.

Looking for a possible stratification of samples based on available metadata, the complete raw panels of detected VOCs and FoodScan physicochemical parameters were inspected by using a Principal component analysis (PCA). As explained by the PCA plot (Supplementary Figure S3), the two plotted principal components that roughly accounted for the 55% of the total distribution, seasonality does not allow for a good cluster separation based on sampling months, whereas a partial division of control versus experimental samples appeared.

The plotted vectors, reported in the PCA biplot graph, evidence a stronger contribution of VOCs in determining the sample placement onto the quadrants. Some fatty acids from physicochemical analysis also contributed (C18:2 n6 trans and C15:1 in the first quadrant).

**Figure 2:**
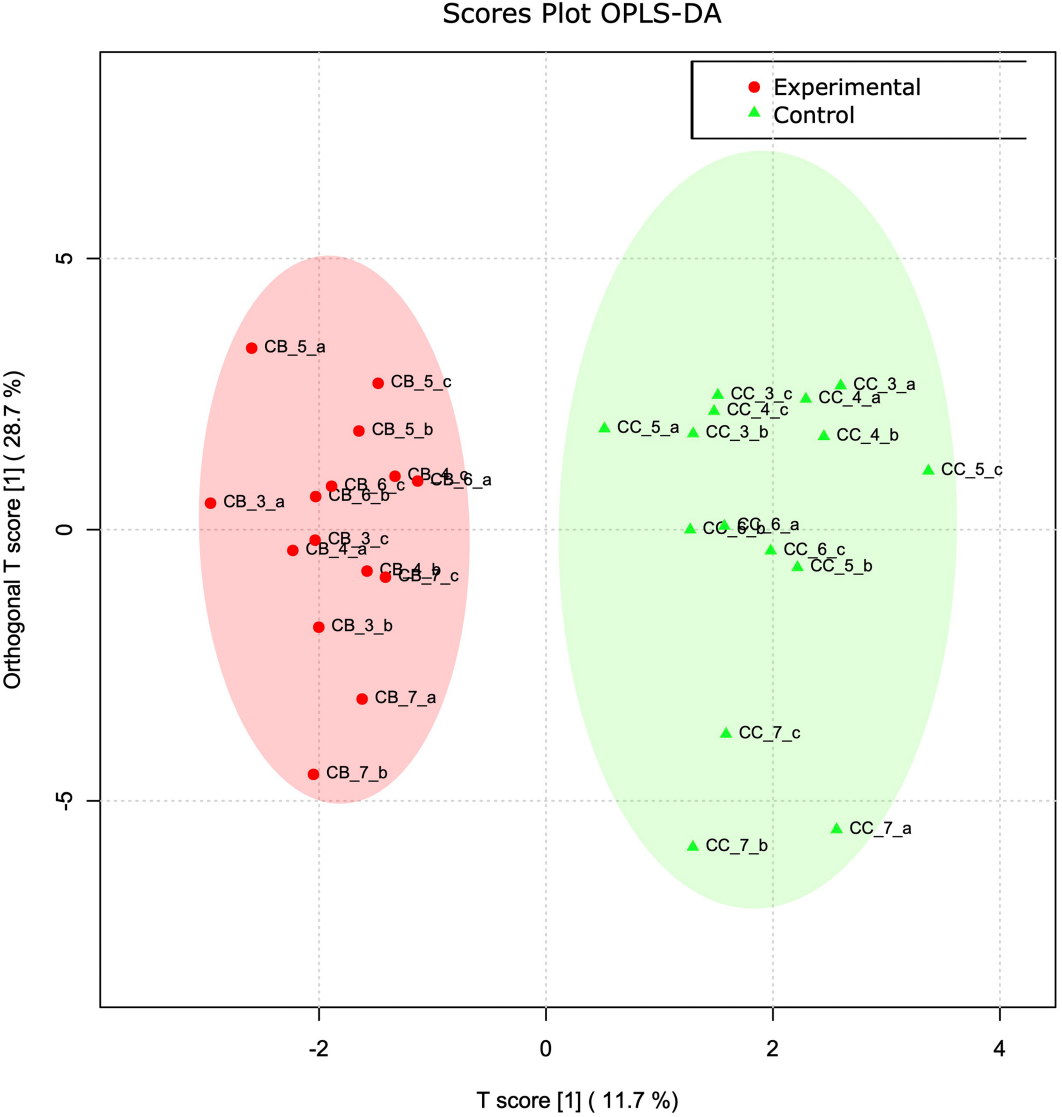
VOC OPLS-DA analysis. OPLS-DA analyses based on VOC abundance normalized matrix. T score and orthogonal T score have been used to plot samples onto a bidimensional graph. Red and green colour indicate experimental and control samples, respectively.

To obtain a better detailing of VOC and FoodScan variable contribution as separate matrices, sample variables from the two analyses were singly analyzed in an Orthogonal Projections to Latent Structures Discriminant Analysis (OPLS-DA) regression analysis. As a matter of fact, the two plots clearly show how the two groups belong to distinct plotted clouds. By separating predictive from non-predictive variation, the resulted VOC T-score and its orthogonal derived values totally accounts for the 40% of the distribution and precisely describe approximately the 29% and 12% on Y and X, respectively (Figure 2). To statistically support this evidence, a permutation analysis of one predicted (p1) and three orthogonal components (o1, o2, o3) that practically relies in the measurement of the observed cross-validated R2X, R2Y, and Q2 coefficients is reported in Supplementary Figure S3.

More in detail panel A of Supplementary Figure S4 reported the most contributing variables, based on the “scores of variable importance on projection” (VIP scores). This weighted sum of OPLS-DA loading squares considers the amount of explained Y-variable in each dimension. 2-Octene, p-cymene, o-cymene, trans-carane, and carane were the most contributing VOCs in terms of VIP score.

**Figure 3:**
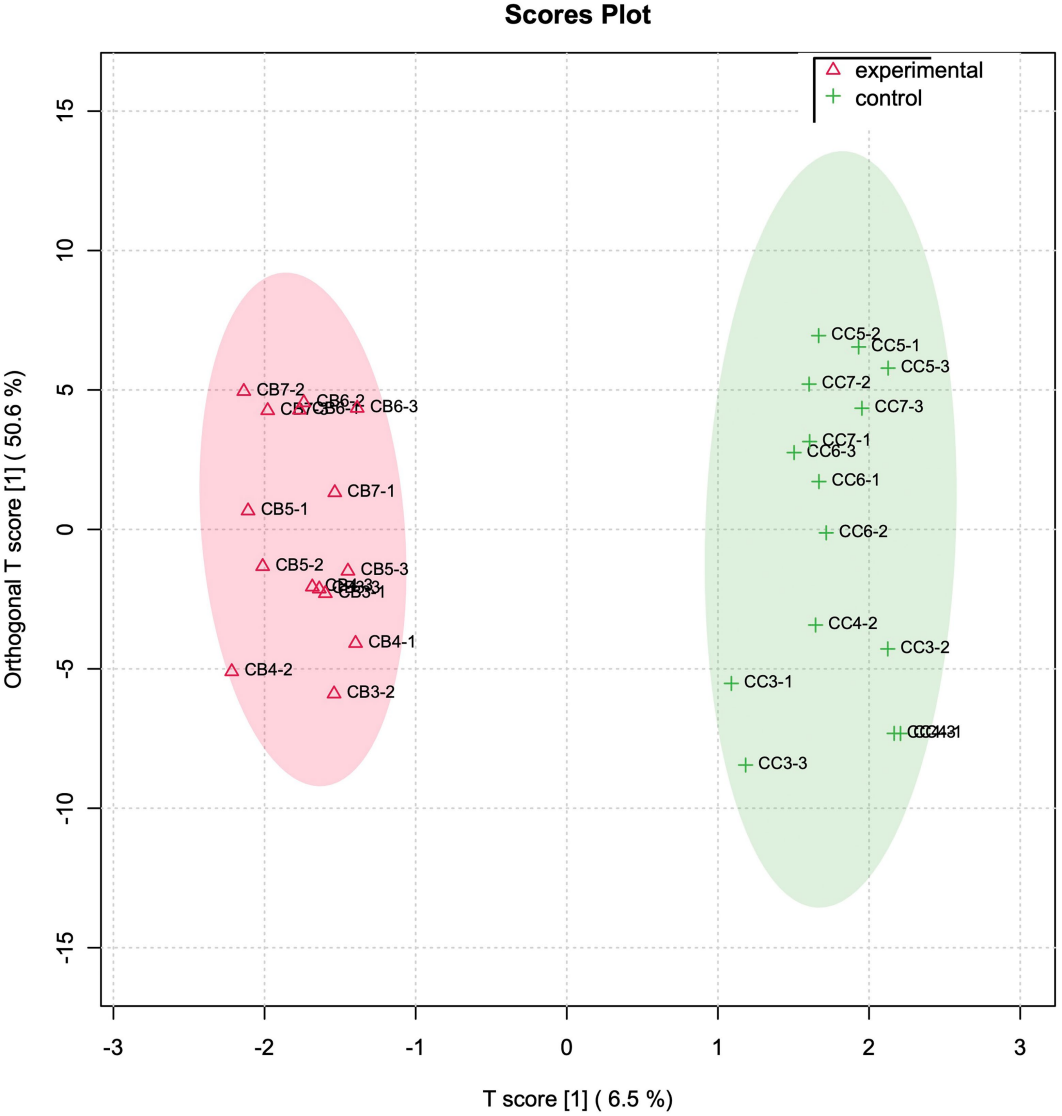
Physicochemical variables OPLS-DA analysis. OPLS-DA analyses based on FoodScan normalized variable matrix. T and orthogonal T score have been used to plot samples onto a bidimensional graph. Red and green colour indicate experimental and control samples, respectively.

In parallel, FoodScan detected cheese variables were investigated by made use of the same multivariate model approach. In this case, the OPLSDA plot reveals some interesting physicochemical variables that much more than other contributed in separating the experimental and control groups (Figure 3). The most contributing FoodScan discriminating variables were evidenced at the top of OPLS-DA VIP score graph (Supplementary Figure S5) and precisely are erucic acid (C22:1 n9), octadecanoic acid (C18:0), polyphenols, tetradecenoic acid (C14:1), octadecatrienoic acid (C18:3 n3), eicosanoic acid (C20:0), and cetoleic acid (C20:1 n9).

**Figure 4:**
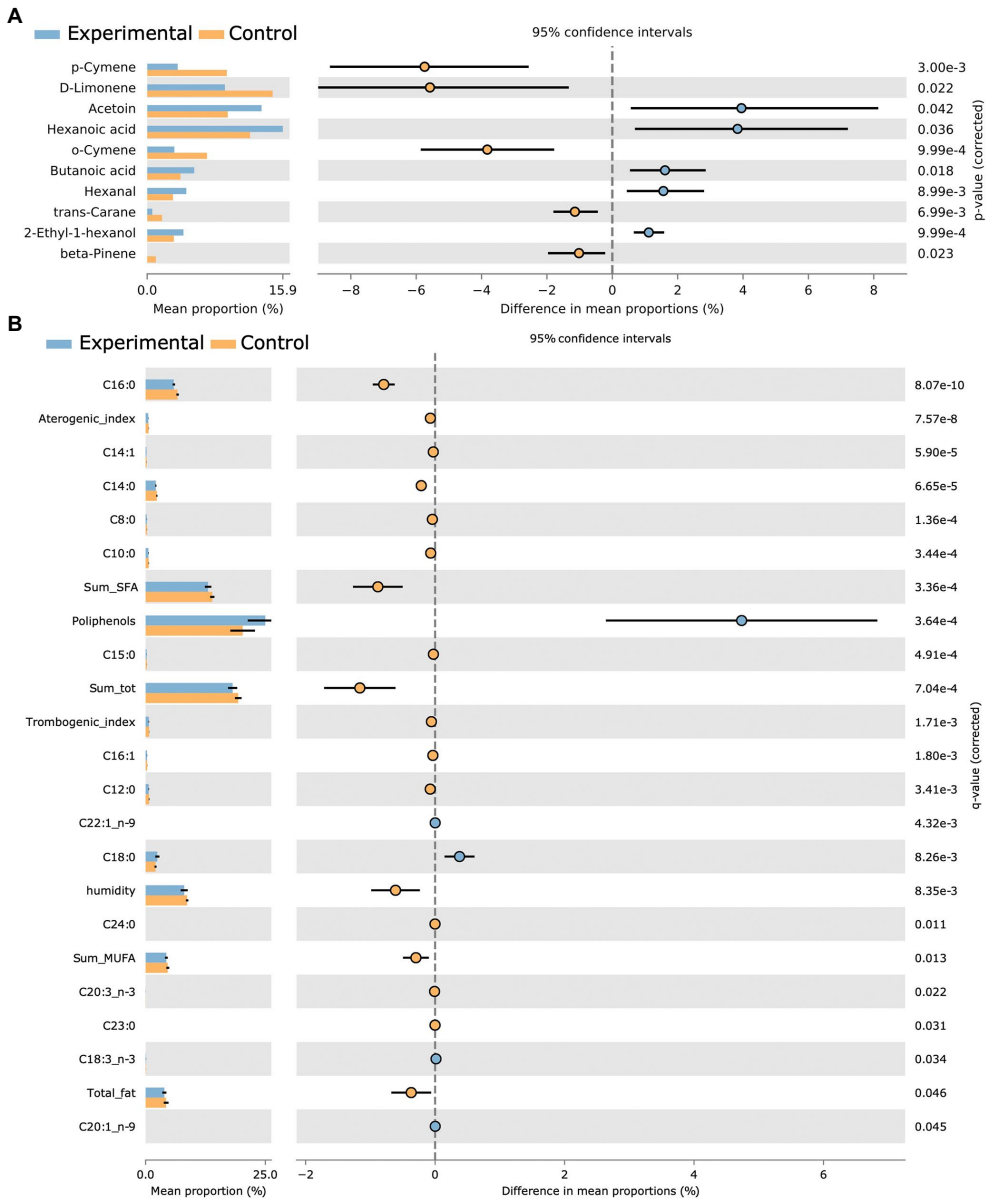
Extended error bars reporting White’s test BH corrected statistically significant variables. Volatile mean proportions (corrected *p* < 0.05) are reported for experimental (blue) and control (orange) groups, together with differences in mean proportions for VOCs **(A)** and physicochemical foodScan variables **(B)**.

To inspect the significance of VOC with a dedicated two group statistics, we made use of a non-parametric corrected (BH) White’s test. The difference in mean proportions was meaningful of different abundance in experimental versus control samples. Acetoin, hexanoic acid, butanoic acid, hexanal, and 2-ethyl-1-hexanol were detected to be higher in experimental group, whereas p-cymene, o-cymene, D-limonene, trans-carane, and beta pinene resulted to be increased in the control group. Figure 4 resumes the mean proportions together with their differences in control and treated groups (corrected *p* < 0.05). The VOC set from OPLS-DA analysis quite totally overlapped with the variables resulted after the White’s non-parametric test application.

Among FoodScan detected fatty acids, C18:0, C18:3 n3, C20:1 n9, and C22:1 n9 were significantly higher in experimental samples.

The two sets of significant variables (VOC and physicochemical parameters from FoodScan) were merged in a single matrix that was used as input for a subsequent correlation analysis (corrected Person’s correlation test). The analysis returns few interesting positive and negative statistically significant correlations.

**Figure 5:**
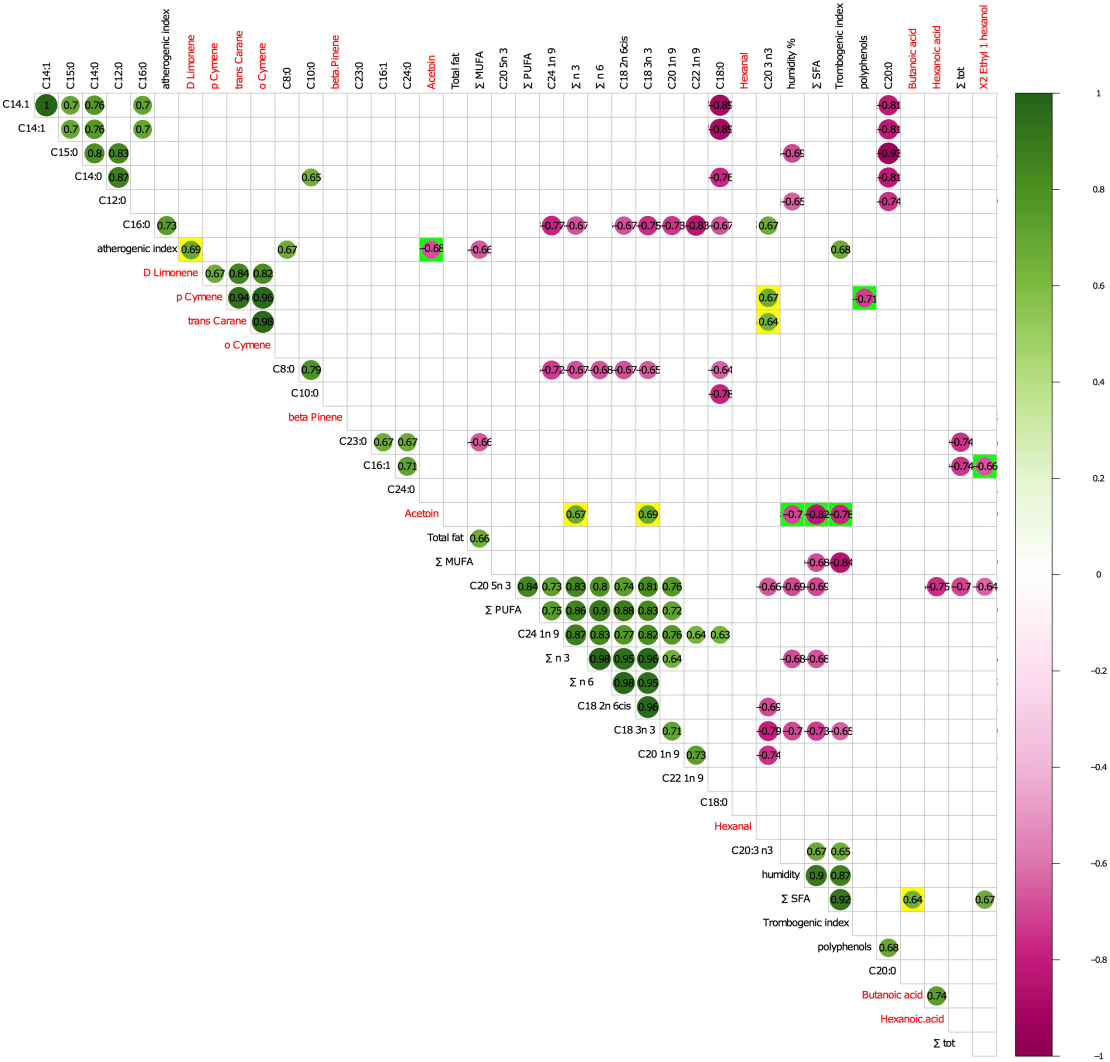
Person correlation analysis between statistically significant VOCs and physicochemicals FoodScan detected parameters. FoodScan physicochemical parameters and volatile organic compounds that significantly differed in the control and experimental groups were correlated with Pearson’s test and only statically significant comparison were reported in the figure. Red and blue fonts mark VOCs and FoodScan parameters, respectively. Following the coloured scale bar, green and purple colours indicate positive and negative correlation, respectively. At a most immediate glance, the two-group paired variables were highlighted by adding yellow or green background colours for positive and negative correlation, respectively.

More in details, as evidenced in Figure 5, the atherogenic index (relative to the monounsaturated fatty acids) positively correlated with D-Limonene and negatively with acetoin. In turn, acetoin positively correlated with polyunsaturated fatty acids of n3 series (∑ n3) and alpha-linolenic acid (C18:3n 3) and negatively with thrombogenic index, percentage of humidity, and sum ∑ SFA.

Both p-cymene and trans-carane positively correlated with C20:3 n3, whereas the first negatively correlated with polyphenols. Finally, X2 Ethyl 1 hexanol and butanoic acid were negatively correlated with Hexadecenoic acid (C16:1) and ∑ SFA, respectively.

## 4. Discussion

In the present study, the evaluation of the effects of dietary olive cake as feed supplementation of lactating dairy cows on fatty acid composition, volatile organic compounds, and microbiological profiles of Provola cheese was performed. Control and experimental Provola cheeses, monthly sampled from March to July 2021, were subjected to physicochemical, microbiological, meta-taxonomic (16S rRNA), and metabolomics (VOCs) analyses.

Overall, microbiological data revealed no significant effects of experimental diet on cultivable microbiota. We hypothesize that in addition to the shared milking, cheese-making, the starter cultures contributed to concealing the effect of diet strategy. However, the slight mean decrease of Enterococci and coagulase-positive staphylococci groups in experimental cheeses could be attributed to polyphenols content of olive cake, which can exert an inhibitory effect on microorganisms (Bonanno et al., 2019). In addition, Milutinović et al. (2021) demonstrated the inhibitory effect of polyphenols against several pathogens, including *E. faecalis*. These findings were almost confirmed by the16S taxa assignment, where really few evidence emerged. Only one statistically significant taxon, i.e., Proteobacteria, was found to be decreased in experimental sample during the summer season (*p* < 0.05). Although this phylum abundance is not supported by statistical significance at lower taxonomic levels, it could reasonably involve the Chromohalobacter and Moraxellaceae taxa, embed as minor contributors in cheeses, most largely present in both the equipment and environments from the production area (Calasso et al., 2016; Haastrup et al., 2018; Gaglio et al., 2021). Within the Moraxellaceae family, the presence of psychrotrophic bacteria genus *Acinetobacter*, detected as a core taxon, by 16S rRNA analysis, could be linked to proteases milk spoilage components in un-ripened cheese (Marino et al., 2019). The alpha and the beta diversities, estimated based on various computed distances, did not show neither a divergent behavior of sample collapsed by fed groups, nor a separation by month/season.

Looking at the physicochemical variables and volatilome profiles, the principal component data (VOCs and FoodScan detected variables) highlighted a better stratification of samples based on fed groups rather than month or seasonal grouping. In addition, the OPLS-DA cross-validation data provided insights into experimental versus experimental groups, based on high-dimensional spectral measurements from GC–MS analysis. In fact, a total of 5 VOCs plus 6 fatty acids and polyphenols among FoodScan detected parameters were achieved. Moreover, VOC and physicochemical data matrices were singularly used in a dedicated pairwise White’s corrected non-parametric statistical tests that allowed the identification of those variables that significatively diverged between control and experimental groups. Our results are consistent with previous studies indicating how dietary supplementation with ingredients containing high oil content produced higher levels of C18:0, C18:3 n3, C20:1 n9, and C22:1 n9 (Lashkari et al., 2020). In particular, the high levels of stearic acid in the experimental group are meaningful of an increased biohydrogenation activity involving microbial MUFA and PUFA (Moate et al., 2014; Chiofalo et al., 2020), linked with animal supplementation with dried and pitted olive cake enriched in unsaturated C18 fatty acid.

We found statistically significant decreased levels of different terpene concentrations including D-limonene, p-cymene, o-cymene, trans-carane, and beta pinene in experimental samples, whereas polyphenols, hexanoic acid, butanoic acid, and acetoin were increased in the same samples.

These lesser detected levels may result from a lower quantitative of ingested hay, as replaced by olive cake (8%). Moreover, we can speculate on the possibility that cow mixed rumen bacteria lead to terpene degradation. Bioavailable terpenes would thus result as the product of the activity of different bacteria consortia and, in a dose-dependent manner, rumen population showed also to have different sensitivity to specific essential oil combinations mainly including medicinal herbs (Poulopoulou and Hadjigeorgiou, 2021). A batch of evidence in literature supports the *in vitro* degradation of terpenes as a result of the exposure to caprine rumen bacteria and lactic acid bacteria used during the cheese-making process (Belviso et al., 2011; Ianni and Martino, 2020). In line with this evidence, the slightly higher microbial counts of LAB in the experimental cheese samples could explain the terpene degradation, and thus the probably effect of experimental supplementation on rumen microbiota.

The correlation analysis of VOC versus FoodScan parameters in cheeses evidenced how, among decreased terpenes, D-limonene levels in experimental samples positively correlated with the atherogenic index (AI), a parameter describing the impact that single fatty acids have on human health. Oppositely, increased levels of acetoin, recognized as a determining factor of soft cheese flavor, were negatively correlated with thrombogenic index and atherogenic indices which were both significantly decreased in experimental samples.

Commonly present in various fresh cheeses, the acetoin presence, as the major volatile component of pasteurized milk cheeses, indicates an upregulation of lactose and citrate metabolisms (Buchin et al., 1998), mainly derived from *Lactococcus* and *Leuconostoc* genera which are primarily responsible for the production of its precursor diacetyl (Dopieralska et al., 2020) and from LAB fermentation (Chen et al., 2017; Dan et al., 2017; Picon et al., 2019).

Being the main contributors of the overall cheese odor of many cheeses, butanoic and hexanoic acids, known for their capacity to impart a cheesy and rancid flavor during ripening (Zabaleta et al., 2016), were both high in experimental cheeses.

As a consequence of increased free fatty acids (FFAs) levels in experimental cheeses, an enhanced lipolytic activity would sustain their increased level in this fresh un-ripened cheese.

2-Ethyl 1 hexanol also significantly increased in our experimental cheeses. It is principally derived from bacterial metabolism, most probably attributable to the Enterobacteriaceae metabolism, that has frequently been found during cheese manufacture (Chaves-López et al., 2006). This VOC imparts a fruity, green, cucumber odor (Morales et al., 2010), and being also ascribed to possible pollution in cheese-making environment (Fay et al., 1999) or linked to air pollutants (Wakayama et al., 2019), its presence needs to be deepened.

The significantly higher polyphenols content of experimental Provola cheeses is another hallmark feature of this dairy food. The increase of bioactive molecules has been related to animal performance as well as to technological, nutritional, and health food properties (Ponte et al., 2022).

## 5. Conclusion

In this study, feeding the dairy cows with olive cake supplementation allowed to achieve several advantages such as an improved product quality in terms of polyphenols, PUFA, and proteins content. In addition, an improvement of the microbiological quality, linked to a decrease of some spoilage-related microbial groups and did not affect the core taxa, of the experimental cheeses as well as volatilome composition was achieved. In conclusion, this paper sheds light on the utilization of the olive cake by-product in farming practices as an aimful administration to obtain a more sustainable and safety dairy food product with lower environmental impact.

## Data availability statement

The datasets presented in this study can be found in online repositories. The names of the repository/repositories and accession number(s) can be found in the article/Supplementary material.

## Ethics statement

The animal study was reviewed and approved by Ethical Committee of the Department of Veterinary Science of the University of Messina. Written informed consent was obtained from the owners for the participation of their animals in this study.

## Author contributions

CR and LL conceived and designed the study. NR, VL, GC, and FL performed the experiments. FC, GC, and MA organized the database and performed the statistical analysis. FC, NR, VL, and GB wrote the manuscript. CR, CC, and AP visualized the results. CR, AP, and LL revised this manuscript. All authors contributed to the article and approved the submitted version.

## Funding

This work was supported by funding from the P.O. Fesr Sicilia 2014/2020. Obiettivo Tematico 1 – Ricerca, Sviluppo Tecnologico e Innovazione Obiettivo specifico 1.1 – Incremento dell’attività di innovazione delle imprese Azione 1.1.5 – Sostegno all’avanzamento tecnologico delle imprese attraverso il finanziamento di linee pilota e azioni di validazione precoce dei prodotti e di dimostrazione su larga scala. Project BIOTRAK. Grant number 08SR1091000150-CUP G69J18001000007 (Principal Investigator Luigi Liotta).

## Conflict of interest

NR, AP, CC, and CR declare that they are members of ProBioEtna, a spinoff of the University of Catania, Italy. In addition, the authors declare that they do not have any personal, financial, professional, political, or legal interests with a significant chance of interfering with the performance of their ethical or legal duties.

The remaining authors declare that the research was conducted in the absence of any commercial or financial relationships that could be construed as a potential conflict of interest.

## Publisher’s note

All claims expressed in this article are solely those of the authors and do not necessarily represent those of their affiliated organizations, or those of the publisher, the editors and the reviewers. Any product that may be evaluated in this article, or claim that may be made by its manufacturer, is not guaranteed or endorsed by the publisher.

## Footnotes

^1^ https://www.ncbi.nlm.nih.gov/sra
