# Supplementary material for: Effect of olive by-products feed supplementation on physicochemical and microbiological profile of Provola cheese
Source: Front Microbiol. 2023 Jan 16;14:1112328. doi: 10.3389/fmicb.2023.1112328 (PMC9885796; doi:10.3389/fmicb.2023.1112328)
Supplement: Supplementary file 2 [file Data_Sheet_1.docx]

Supplementary Material

# Supplementary Figures

**Supplementary figure S1.** **Taxa Welch’s test.** a) Boxplot relative to the distribution of the only one statistically different detected phylum in control versus experimental samples. b) Mean relative frequencies and standard deviation for the two compared groups.

**Supplementary figure S2. Unweighted unifrac PCoA relative to experimental and control cheese sample beta diversity.** Tridimensional plot reporting the beta diversity computed by using the dedicated Emperor plugin within the QIIME2 pipeline. Blue and red dots indicate experimental and control samples, respectively. Panel b and c are relative to the obtained distance distribution for the control and experimental groups obtained with a PERMANOVA pairwise test. D) PERMANOVA statistic based on Bray-Curtis distance values.

**Supplementary figure S3. PCA analyses on VOC and physicochemical parameters (Foodscan).** The complete matrix including VOCs and FoodScan detected variables was normalized and used as input for the PCA analyses (panel A) whose principal components are explained in terms of contributing variables in the biplot (panel B).

**Supplementary figure S4. VOC’s OPLS-DA supporting statistics.** A) OPLS-DA variable importance in projection (VIP) plot computed on the complete panel of detected VOCs. Most important metabolite features identified by OPLS-DA (2-Octene, p-cymene, o-cymene, trans-carane and carane) are ranked on the top of the plot. Black and white boxes on right indicate relative concentration of corresponding VOC for samples belonging to control and experimental groups. B) cross validated Q2/R2X/R2Y coefficients produced as a result of a permutation analysis between one predictive (p1) and three orthogonal (o1, o2, o3) components.

**Supplementary Figure S5. FoodScan physicochemical parameter’s OPLS-DA supporting statistics.** A) OPLS-DA variable importance in projection (VIP) plot computed on the complete panel of detected FoodScan physicochemical variable. Most important features identified by OPLS-DA (erucic acid (C22:1 n9), octadecanoic acid (C18:0), polyphenols, tetradecenoic acid (C14:1), octadecatrienoic acid (C18:3 n3), eicosanoic acid (C20:0), cetoleic acid (C20: 1 n9)) are ranked on the top. Black and white boxes on right indicate relative concentration of corresponding VOC for samples belonging to control and experimental groups. B) cross validated Q2/R2X/R2Y coefficients produced as a result of a permutation analysis between one predictive (p1) and four orthogonal (o1, o2, o3, o4) components.

# Supplementary Tables

**Supplementary Table 1.** Least Squares Mean of milk quality from Holstein cows fed with Ctr or Exp diet

|  | | | | p-value | | |
| --- | --- | --- | --- | --- | --- | --- |
| Item | Ctr | Exp | SEM^1^ | Treatment^2^ | Time^3^ | Treatment*Time^4^ |
| Fat, % | 3.60 | 3.70 | 0.09 | 0.21 | 0.07 | 0.34 |
| Protein, % | 3.34 | 3.30 | 0.08 | 0.24 | 0.05 | 0.20 |
| Lactose, % | 4.82 | 4.80 | 0.07 | 0.10 | < 0.05 | 0.25 |
| Casein, % | 2.62 | 2.58 | 0.04 | 0.42 | < 0.05 | 0.47 |
| LOG_10_ SCC, x 1000 cells/ml | 5.39 | 5.17 | 0.06 | 0.15 | 0.80 | 0.98 |
| ^1^ Greatest standard error of the mean; ^2^ Treatment = overall effect of diet (Ctr vs. Exp); ^3^ Time = overall effect of sampling time; ^4^ Treatment*Time = Effect of the interaction Treatment and Time | | | | | | |

**Supplementary Table 2.** Microbiological counts of milk samples.

| Microbial groups | March | | April | | May | | June | | July | |
| --- | --- | --- | --- | --- | --- | --- | --- | --- | --- | --- |
|  | Ctr | Exp | Ctr | Exp | Ctr | Exp | Ctr | Exp | Ctr | Exp |
| Enterobacteriaceae | <1 | <1 | <1 | <1 | <1 | <1 | <1 | <1 | 1.53±0.02 | <1 |
| *E. coli* | <1 | <1 | <1 | <1 | <1 | <1 | <1 | <1 | <1 | <1 |
| Coliforms | 1.30±0.03 | <1 | <1 | <1 | <1 | <1 | <1 | <1 | <1 | <1 |
| Total mesophilic bacteria | 2.81±0.01 | 2.95±0.05 | 1.48±0.07 | 1.92±0.02 | 2.81±0.02 | 2.07±0.04 | 2.43±0.01 | 2.41±0.00 | 2.40±0.02 | 2.33±0.02 |
| *L. monocytogenes* | absent | absent | absent | absent | absent | absent | absent | absent | absent | absent |

Data are presented as mean log cfu/g ± standard deviation, based on 3 replicates.

**Supplementary Table 3.** Cheese physicochemical parameter in Ctr and Exp samples during the experimental period.

|  | March | | April | | May | | June | | July | |  |
| --- | --- | --- | --- | --- | --- | --- | --- | --- | --- | --- | --- |
|  | Exp | Ctr | Exp | Ctr | Exp | Ctr | Exp | Ctr | Exp | Ctr |  |
| Moisture (%) | | 37.34 ± 0.84^D^ | 44.65 ± 1.45^B^ | 37.54 ± 1.00^D^ | 42.21 ± 1.80^C^ | 43.66 ± 0.34^BC^ | 39.18 ± 0.22^D^ | 45.17 ± 0.16^B^ | 43.32 ± 0.23^BC^ | 48.03 ± 0.06^A^ | 44.30 ± 0.29^B^ |
| Proteins (%) | | 25.64 ± 1.75^A^ | 21.97 ± 0.82^D^ | 26.33 ± 1.12^A^ | 23.36 ± 0.32^BCD^ | 23.39 ± 0.32^BCD^ | 25.09 ± 0.48^AB^ | 23.39 ± 0.28^BCD^ | 22.82 ± 0.71^CD^ | 24.70 ± 0.31^ABC^ | 25.83 ± 0.38^A^ |
| Total lipids (%) | | 22.46 ± 1.26^AB^ | 19.78 ± 2.62^BCD^ | 21.77 ± 1.60^ABC^ | 17.87 ± 0.42^D^ | 18.93 ± 0.88^CD^ | 21.84 ± 1.66^ABC^ | 21.44 ± 1.12^ABC^ | 23.05 ± 1.35^A^ | 18.09 ± 0.45^D^ | 22.94 ± 0.52^AB^ |
| Polyphenols (ppm) | | 121.18 ± 9.30^BC^ | 108.85 ± 9.06^CD^ | 130.04 ± 5.95^B^ | 107.02 ± 7.33^CD^ | 92.07 ± 1.91^E^ | 72.11 ± 0.46^F^ | 165.84 ± 7.96^A^ | 100.65 ± 1.33^DE^ | 152.64 ± 3.65^A^ | 112.26 ± 6.38^CD^ |

^A-F^ Different lowercase letters represent significant differences within rows.

# Supplementary Data

**Supplementary data 1.** 16S rRNA raw data
