# Supplementary figures and images for: Effect of olive by-products feed supplementation on physicochemical and microbiological profile of Provola cheese
Source: Front Microbiol. 2023 Jan 16;14:1112328. doi: 10.3389/fmicb.2023.1112328 (PMC9885796; doi:10.3389/fmicb.2023.1112328)

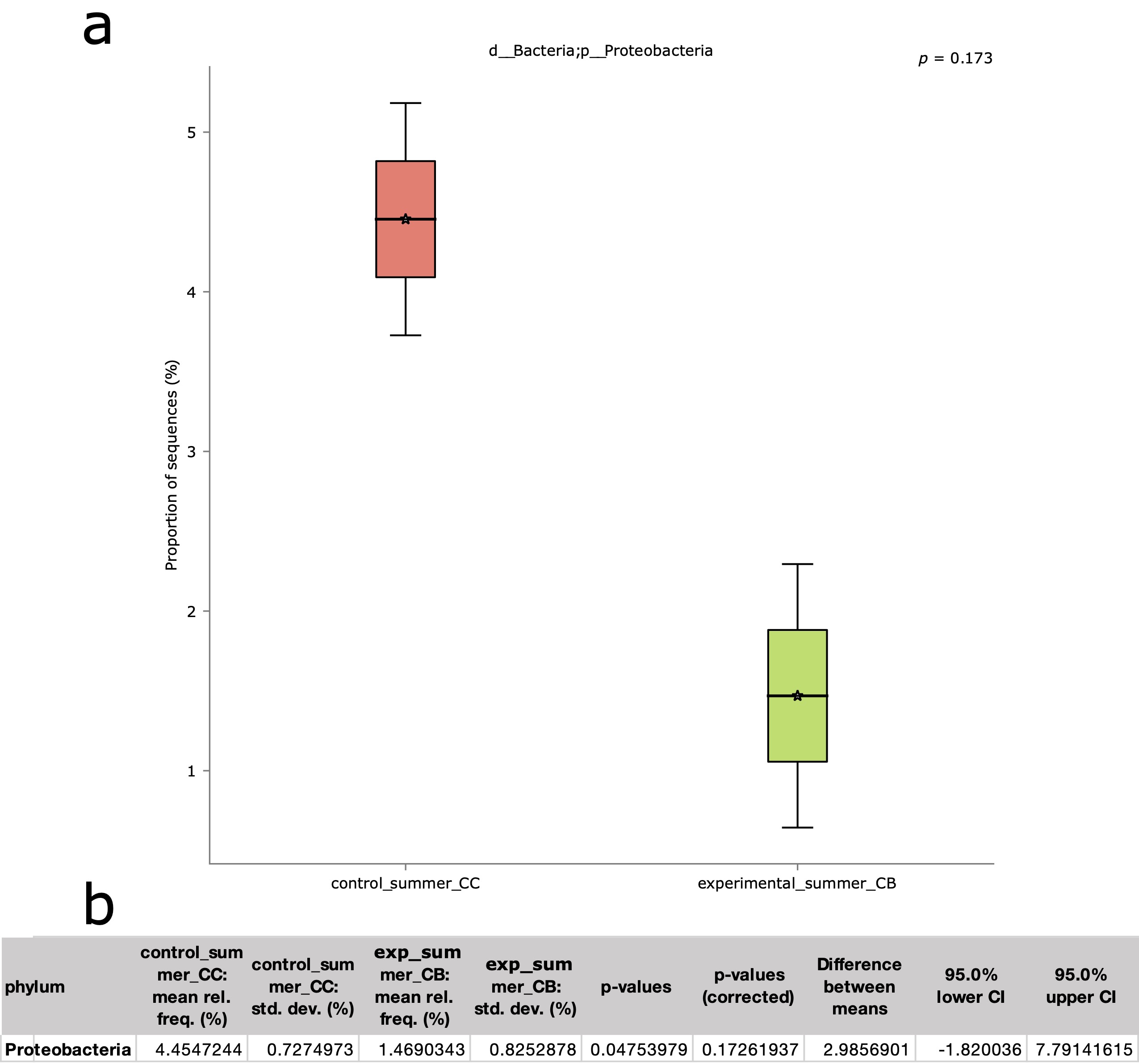

Supplement: Supplementary file 3 [file Image_1.JPEG]

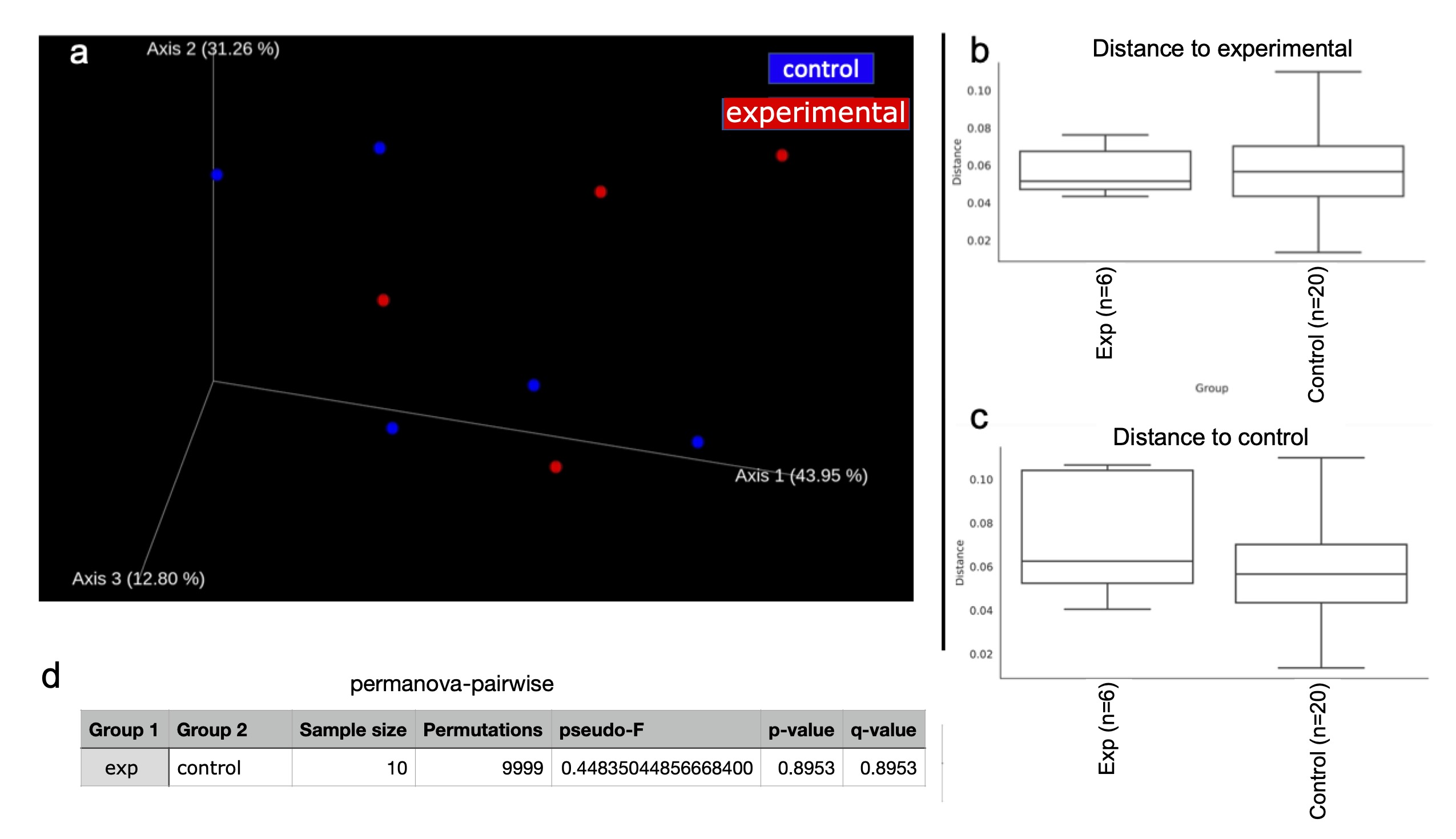

Supplement: Supplementary file 4 [file Image_2.JPEG]

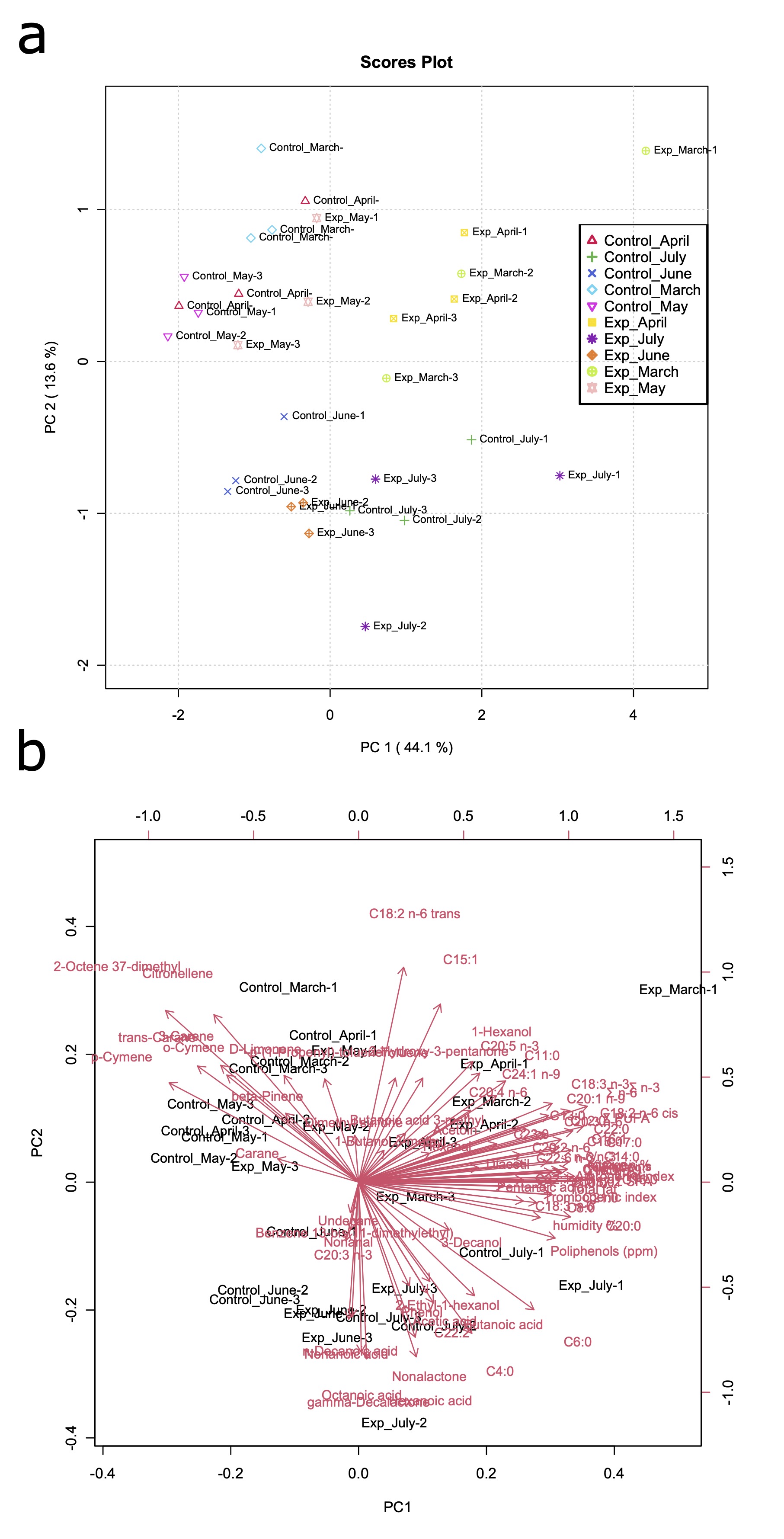

Supplement: Supplementary file 5 [file Image_3.JPEG]

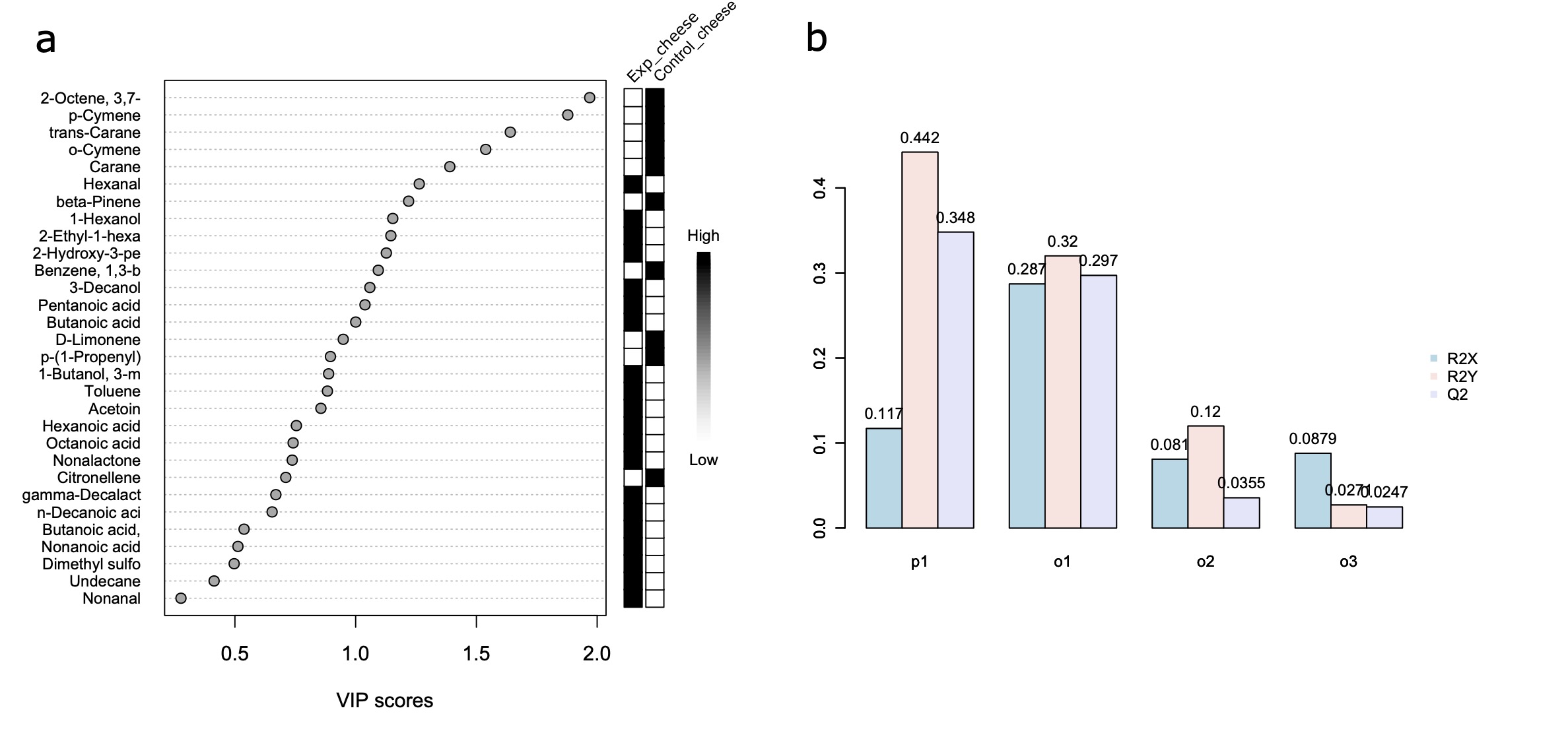

Supplement: Supplementary file 6 [file Image_4.JPEG]

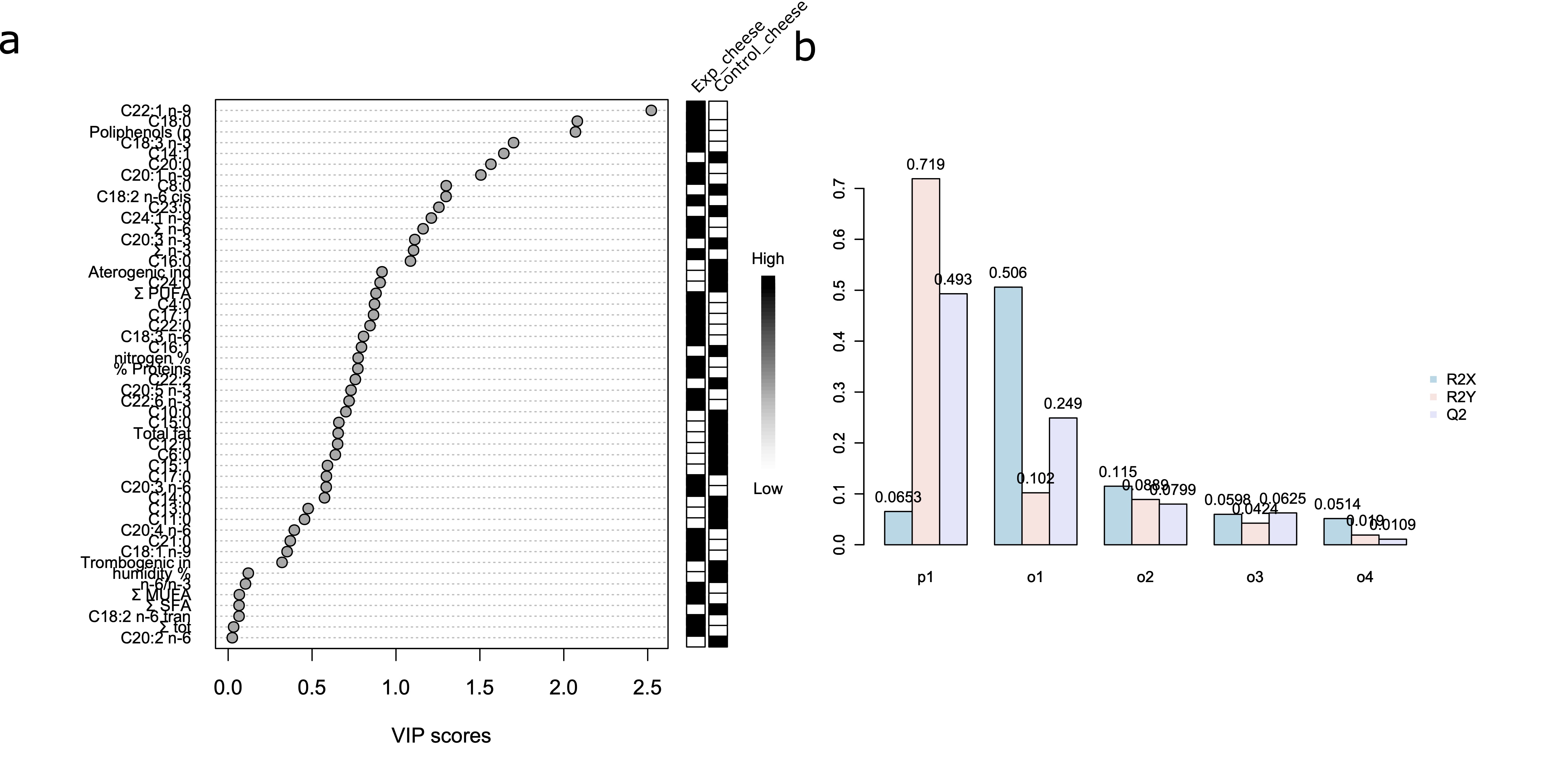

Supplement: Supplementary file 7 [file Image_5.JPEG]
